# Improved clinical outcomes after non-invasive oocyte selection and Day 3 eSET in ICSI patients

**DOI:** 10.1186/s12958-021-00704-5

**Published:** 2021-02-19

**Authors:** Inge Van Vaerenbergh, Tom Adriaenssens, Wim Coucke, Lisbet Van Landuyt, Greta Verheyen, Michaël De Brucker, Michel Camus, Peter Platteau, Michel De Vos, Elien Van Hecke, André Rosenthal, Johan Smitz

**Affiliations:** 1 Follicle Biology Laboratory, Vrije Universiteit Brussel, 1090 Brussels, Belgium; 2 Fertiga, 1090 Brussels, Belgium; 3 Quality of Laboratories, Sciensano, 1050 Brussels, Belgium; 4 Centre for Reproductive Medicine, Universitair Ziekenhuis Brussel, 1090 Brussels, Belgium

**Keywords:** Cumulus cells, Non-invasive, Gene expression, Single embryo transfer, Clinical pregnancy, Live birth

## Abstract

**Background:**

Non-invasive oocyte quality scoring, based on cumulus gene expression analysis, in combination with morphology scoring, can increase the clinical pregnancy (CPR) and live birth rates (LBR) in Day 3 eSET (elective single embryo transfer) ICSI patients. This was first investigated in a pilot study and is now confirmed in a large patient cohort of 633 patients. It was investigated whether CPR, LBR and time-to-pregnancy could be improved by analyzing the gene expression profile of three predictive genes in the cumulus cells, compared to patients with morphology-based embryo selection only.

**Methods:**

A large interventional, non-randomized, assessor-blinded cohort study with 633 ICSI patients was conducted in a tertiary fertility center. Non-PCOS patients, 22–39 years old, with good ovarian reserve, were stimulated with HP-hMG using a GnRH antagonist protocol and planned for fresh Day 3 eSET. The cumulus cells from individually denuded oocytes were ranked by a lab-developed cumulus cell test: qRT-PCR for three predictive genes (*CAMK1D*, *EFNB2* and *SASH1*) and two control genes (*UBC*, *B2M*). The embryo selected for transfer was highest ranked from the pool of morphologically transferable Day 3 embryos. Patients in the control (*n* = 520) and experimental arm (*n* = 113) were compared for clinical pregnancy and live birth, using a weighted generalized linear model, and time-to-pregnancy using Kaplan-Meier curves.

**Results:**

The CPR was 61% in the experimental arm (*n* = 113) vs 29% in the control arm (*n* = 520, *p* < 0.0001). The LBR in the experimental arm (50%) was significantly higher than in the control arm (27%,*p* < 0.0001). Time-to-pregnancy was significantly shortened by 3 transfer cycles independent of the number of embryos available on Day 3 (Kaplan-Meier, *p* < 0.0001).

Cumulus cell tested patients < 35 years (*n* = 65) or ≥ 35 years (*n* = 48) had a CPR of 62 and 60% respectively (ns). For cumulus cell tested patients with 2, 3–4, or > 4 transferable embryos, the CPR was 66, 52, and 67% (ns) respectively, and thus independent of the number of transferable embryos on Day 3.

**Conclusions:**

This study provides further evidence of the clinical usefulness of the non-invasive cumulus cell test over time in a larger patient cohort.

**Trial registration:**

Clinicaltrials.gov, NCT03659786/NCT02962466 (Registered 6Sep2018/11Nov2016, retrospectively registered.

**Supplementary Information:**

The online version contains supplementary material available at 10.1186/s12958-021-00704-5.

## Background

Assisted reproductive technology (ART) has been used for the last four decades to overcome infertility. Conventional in-vitro fertilization (IVF) or intracytoplasmic sperm injection (ICSI) with a fresh or frozen single (SET) or double (DET) embryo transfer on Day 3, or a fresh or frozen SET on Day 5 are most commonly used.

Despite all technological improvements over the last 20 years, the majority of the patients still need two or three ART treatment cycles before taking a baby home [13]. Success rates of ART cycles are generally expressed as clinical pregnancy rates (CPR) at week 5–6 of gestation, ongoing clinical pregnancy rate (OPR) at week 10–11 of gestation, or live birth rate (LBR). Success rates depend on many clinical factors of the female and male undergoing IVF or IVF-ICSI, and a specific ART treatment is chosen in a patient-tailored manner. Age and ovarian reserve are prominent clinical factors influencing ART outcomes [4, 10]. Success rates also differ significantly depending on the day of transfer [20, 25]. Generally, CPR and LBR rates for a fresh Day 3 SET are between 27 and 35% and 23–29% in Europe [5, 7, 22], respectively, for women between 22 and 38 of age with good ovarian reserve and for first or second ranked ICSI cycles. For a same patient profile, OPR and LBR for a fresh Day 5 SET are reported to be around 40–45%, and 35–40%, respectively (European MEGASET study, [15]). Freeze-all strategies may yield higher success rates due to the ability to prepare the endometrium systematically for the embryo transfer, notably in hyper-responders and in patients undergoing PGT-A [27, 35]. However, a new RCT showed no difference in OPR and LBR between freeze-all Day 5 transfer and a fresh Day 5 transfer [33]. In highly selected cohorts of young patients, success rates of 50–60% per blastocyst transfer have been reported with a “freeze-all” strategy in combination with preimplantation genetic testing for aneuploidy screening (PGT-A) [24, 29].

During the last decades, there is an increasing trend towards applying single embryo transfer because multiple embryo transfer increase the risk for multiple pregnancy, thereby increasing the risk of complications, prematurity and major malformations like cerebral palsy, neural tube defects and esophageal atresia [6, 26]. However, still 65% of embryo transfers in Europe [12] and 60% in US [14] are double or even triple embryo transfers.

However, overall success rates in the entire ART population across all ages and conditions remain low. LBR per oocyte retrieval in Europe is only 20–22% [12]. In the US, LBR per initiated cycle is 22% on average [14]. Over the last decade, a decline in live birth after ART has even been observed [19].

As such, there is a need to develop and validate non-invasive technologies that focus on eSET and could increase outcomes in IVF-ICSI procedures. Cumulus cells, surrounding the oocytes, are important key contributors towards oocyte development, paving the way to using gene expression in cumulus cells as potential biomarkers. Over the years, more than one hundred genes have been identified and linked with embryo development, pregnancy and live birth ([3, 8, 16, 18, 23]; for a recent review [32]). However, only a few gene sets have been studied in more detail. The expression of three genes in cumulus cells (*PTGS2*, *CAMK1D*, *HAS2*) was associated with embryo development to the blastocyst stage [30]. In a previous pilot study by our group, three genes (*SASH1*, *CAMK1D* and *EFNB2*) were used successfully to assess oocyte quality in an algorithm (formerly known as Corona Test, [1]). The ranking of the oocyte according to quality -based on the cumulus cell test result- was used to guide the selection of an embryo for transfer on Day 3 and led to significantly increased CPR and LBR in a prospective clinical study [1]. Interestingly, *CAMKD1* was confirmed by others as a biomarker for oocyte development and as a predictor of success rate in ART [30].

### Study objectives

The primary objective was to investigate whether, in a larger patient cohort of patients with a Day 3 eSET, CPR could be increased by analyzing the gene expression profile of 3 predictive genes in the cumulus cells compared to patients with morphology-based embryo selection only. Secondary endpoints were 1. LBR, 2. CPR and LBR in subgroups according to age and number of transferable embryos, and 3. time-to-pregnancy.

## Methods

### Study participants & inclusion/exclusion criteria

Women up to 39 years old, stimulated with HP-hMG (Menopur®, Ferring Pharmaceuticals, St. Prex, Switzerland), in a GnRH antagonist protocol, who presented at a tertiary referral hospital between October 2013 and April 2019, scheduled for ICSI and fresh single embryo transfer on Day 3, were eligible for this interventional, non-randomized, assessor-blinded cohort study with one experimental arm and one control arm. Patients with polycystic ovary syndrome (PCOS, Rotterdam 2003 criteria [28]) and/or severe male infertility were excluded from the study. There was no statistical difference in infertility indication (male, female or mixed infertility) between the arms of the study. Patients enrolled in other studies or scheduled for PGT (pre-implantation genetic testing) were also excluded. Patients with only one or no transferable embryo on Day 3 were considered drop-outs.

### Design of the predictive model

The design of the predictive model and the selection of the three genes was described before in a manuscript reporting the results of our pilot study [1]. In brief, more than 140 Affymetrix arrays on individual cumulus cells (CC) of SET ICSI patients were used for transcriptome analysis with embryo development and live birth as endpoints. The different microarray analyses revealed a multitude of potential embryo development and pregnancy predicting genes (unpublished data). From these lists, 23 predictive genes were validated over time in independent biological replicates using qRT-PCR, with a focus on genes predicting pregnancy. Several of these qPCR studies were published [2, 36–38]. While the expression of many genes could be related to oocyte competence, the two main challenges were: finding the strongest combination of genes and finding a model predictive for live birth. This validation strategy together with intrapatient comparisons have led to the current pregnancy prediction model (AUC 0,8081; accuracy 80%). EFNB2, SASH1, and CAMK1D have been linked to cell expansion [9], the Toll-like receptor 4 pathway [11], and the calcium pathway, respectively. In our study, EFNB2 and SASH1 expression were positive correlated and CAMK1D exon 1 expression was negatively correlated with clinical pregnancy ([1], see Supplementary Figure 1).

This mathematical model, comprising only gene expression results for predicting clinical pregnancy and live birth, was previously named Corona Test [1] and in this manuscript is named cumulus cell test. This test will be offered under the name “Aurora Test” (The name was changed because of the current Corona virus pandemic).

### Collection and expression analysis of cumulus cells

Cumulus cells (approx. 1000–30,000 cells/oocyte, extrapolated from total RNA measured with BioAnalyzer Pico Chip, Agilent) were collected after single-oocyte denudation using Cumulase (Origio, CooperSurgical) [34]. The individual oocyte denudation procedure (on average 8 oocytes per cycle) in the experimental arm required 15–30 min extra handling time compared to grouped oocyte denudation, depending on the number of oocytes per cycle. Within 4 h after oocyte retrieval, the cumulus cells were removed and ICSI was performed immediately thereafter. Cumulus samples of all fertilized oocytes were analyzed prospectively at the Follicle Biology laboratory of Vrije Universiteit Brussel - Universitair Ziekenhuis Brussel, Belgium, for all 113 patients in the experimental arm on Day 1 or 2 after oocyte retrieval. The lab performing the cumulus cell test was blinded for the morphological quality scoring of the embryos. Total RNA extraction on cumulus cells was performed with the RNeasy Micro kit (Qiagen, The Netherlands) on the Qiacube (Qiagen) and reversed transcription was performed with the iScript cDNA synthesis kit (BioRad, Belgium). cDNA was frozen at − 80 °C until further qPCR analysis. The three specific genes (EFNB2, SASH1, and CAMK1D) were analyzed together with two endogenous control genes, UBC and B2M. The mean of B2M and UBC expression was used as the normalization factor. All PCR quantifications (LC480, Roche Diagnostics) were performed in triplicate (for the specific genes EFNB2, CAMK1D and SASH1) or duplicate (for the endogenous controls B2M and UBC) for the samples, and in triplicate for the calibrators and negative controls. The average coefficient of variation was < 0,1 Cp for all assays applied. The mean laboratory turnaround time from the start of the sample processing by nucleic acid extraction and qRT-PCR up to the completion of the final report of the analysis was on average 8 h.

### Embryo selection using the oocyte ranking

The normalized expression levels of the three genes were used to calculate a cumulus cell test ranking. Ranking data were reported to the embryology lab in the morning of Day 3. Embryos underwent the standard morphological evaluation comprising the scoring of fertilization, Day 2 embryo quality and full embryo grading on Day 3, as described previously [31]. Among the embryos that were morphologically eligible for transfer, the embryo with the highest cumulus cell test rank was selected by the embryologist for an eSET on Day 3.

### Definition of outcomes

The primary outcome measure was clinical pregnancy defined as the ultrasonographic visualization of a fetal sac at week 7 or later with normal fetal heartbeat. It also includes ectopic pregnancy [40, 41].

The secondary outcome measure was live birth defined as the complete expulsion or extraction from a woman of a product of fertilization, after 22 completed weeks of gestational age; which, after such separation, breathes or shows any other evidence of life, such as heart beat, umbilical cord pulsation or definite movement of voluntary muscles, irrespective of whether the umbilical cord has been cut or the placenta is attached [41].

Another secondary outcome measure was time-to-pregnancy defined as the time taken to establish a pregnancy, measured in months or in numbers of menstrual cycles [41]. In case of artificial ART cycles, it was measured in number of embryo transfer cycles.

### Statistical analysis

An absolute increase of CPR of 25% points in the experimental arm over the control arm was assumed using a weighted generalized linear model for CPR and LBR, in which the weights given to the control patients were proportional to the number of patients in the experimental arm with the cumulus cell test in each corresponding group based on transferable embryos and age category. Sample size calculation showed that, if we considered a 95% power, and 20% variability in a two-sided test, because the morphological evaluation is performed by several embryologists, 107 informative patients were needed in the experimental arm, after all drop-outs, to show a 25% increase of CPR after fresh eSET. To prevent eventual confounding, all statistical analyses were stratified by age of the woman, and number of transferable embryos available on Day 3.

Patient characteristics were compared using the Wilcoxon rank sum test.

Information on cumulative CPR and LBR was obtained for all frozen embryo transfers. When considering the fresh and frozen transfers within 1 year from the start of treatment, the time-to-pregnancy was compared between the two arms using Kaplan-Meier curves. For this analysis, 113 exactly matched controls (matched for age, number of transferable embryos and closest in time to the treated case), out of the available 520 controls needed to be used.

All calculations were performed in S-plus 8.0 for Linux or GraphPad Prism, *p* values of <.05 were considered to be significant.

## Results

### Patient flow

**Fig. 1:**
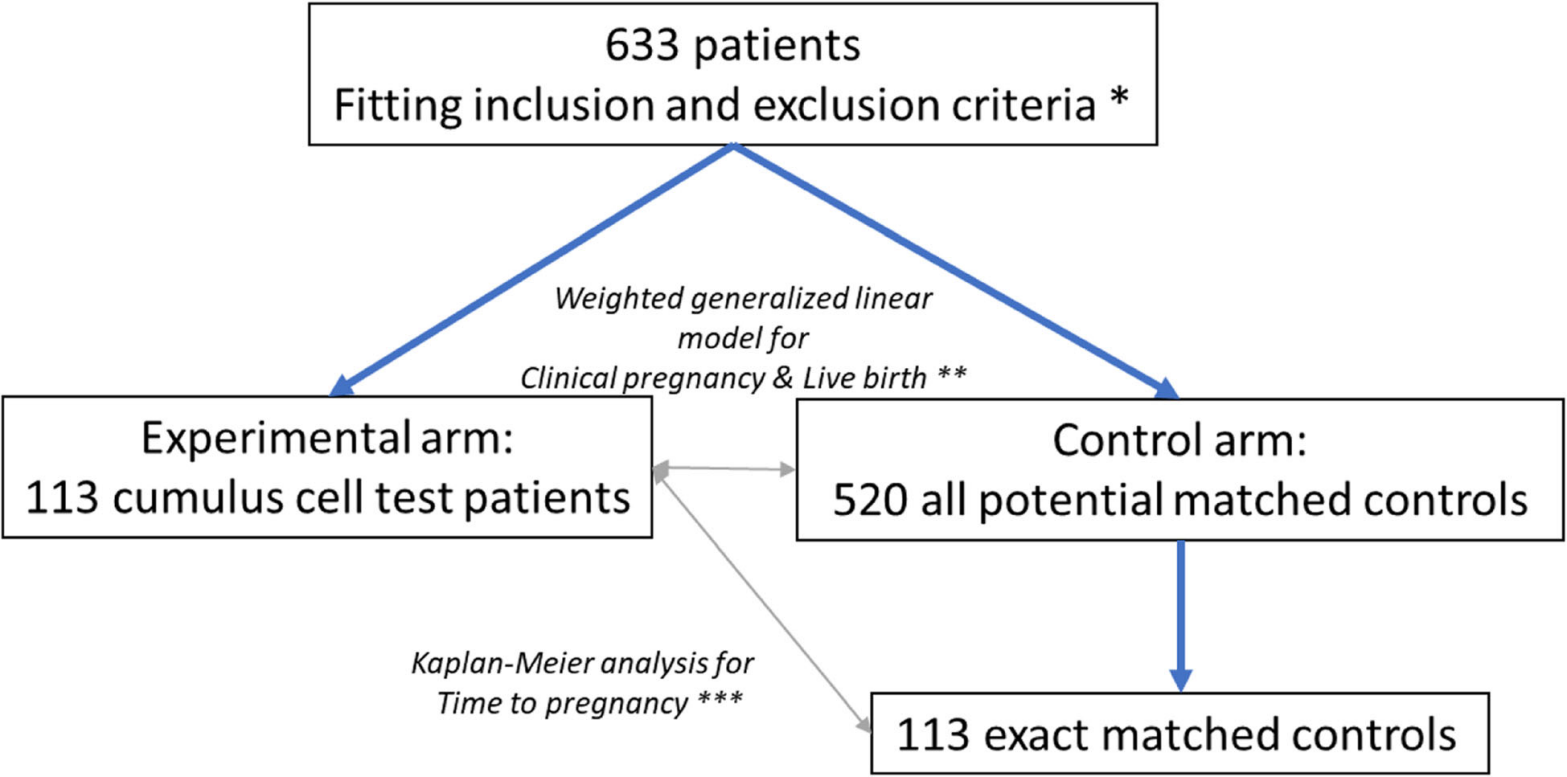
Patient flow. *Patients eligible for the cumulus cell test study: Not recruited for any other study, stimulated with HP-hMG in a GnRH antagonist protocol from Day 6, fresh eSET, scheduled for ICSI, up to 39 years old, with at least 2 transferable embryos on Day 3, with follow up data (up to clinical pregnancy) Excluded: PGD/PGS, TESE, FNA, PCOS, known poor ovarian response. **: A weighted generalized linear model was used with weighted averages to stratify the populations for age and number of transferable embryos on Day 3. *** to prevent potential confounding factors 113 exact matched controls nearest in time to the experimental case were retained for the Kaplan-Meier analysis

This was an interventional, non-randomized, assessor-blinded cohort study with 633 patients, 113 patients in the experimental arm with the cumulus cell test and 520 patients in the control arm (Fig. 1). The study was designed to validate a 25% increase in CPR by ranking transferable embryos based on gene expression in cumulus cells. Secondary endpoint was to achieve a significant increase in LBR in the experimental arm. The number of controls was 4.7 times higher than the number of cases to ensure reliable data for CPR and LBR in the control arm.

The strict inclusion and exclusion criteria considerably limited the number of patients eligible for this study. The most important factors limiting patient inclusion were: (i) one specific stimulation protocol (GnRH antagonist with HP-hMG), and (ii) one specific embryo transfer regime (fresh eSET Day 3). Patients enrolled in other clinical studies performed at Universitair Ziekenhuis Brussel were excluded from this study.

### Patient characteristics

**Fig. 2:**
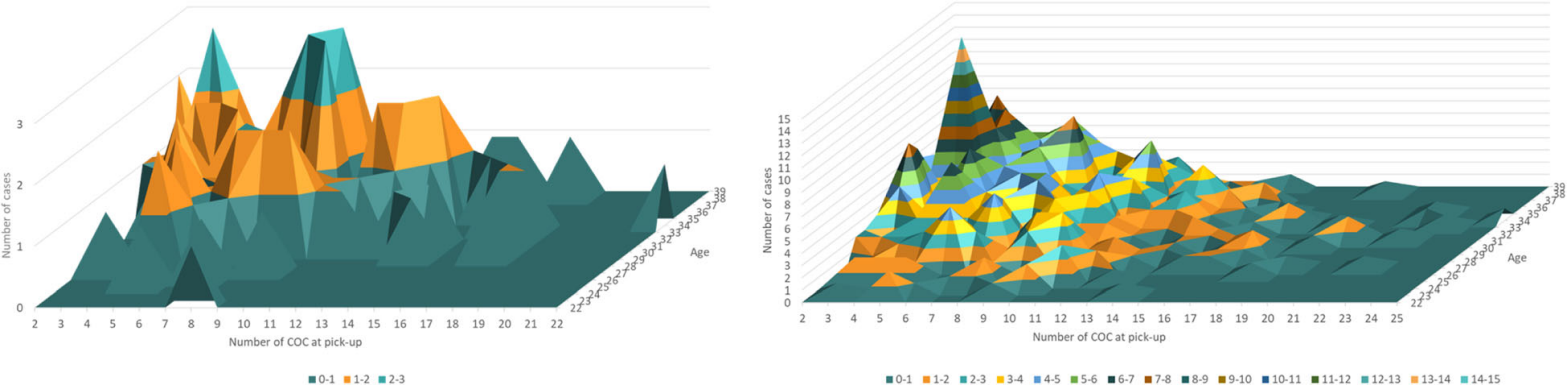
Three-dimensional histogram of the experimental arm (*n* = 113, left side) and control arm (*n* = 520, right side) depicting age vs the number of COC at oocyte pick-up (number of cases per data point are depicted with different colours)

**Fig. 3:**
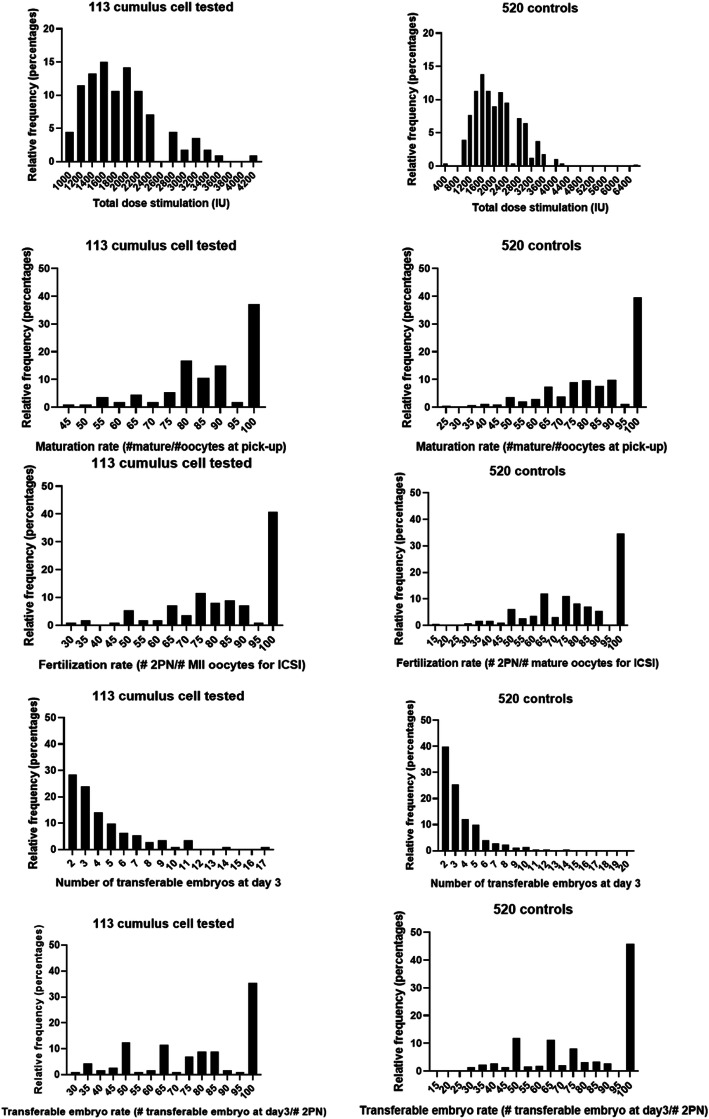
Histograms of total stimulation dose (IU), maturation rate (%), fertilization rate (%), number of transferable embryos (n) at Day 3 and transferable embryo rate (number of transferable embryos at Day 3 divided by the number of fertilized embryos, %) for the experimental arm (*n* = 113) and the control arm (*n* = 520). Distributions are shown as relative frequency distributions (%)

The clinical patient characteristics in the experimental arm and the control arm were similar for most parameters except for the total stimulation dose and the number of transferable embryos on Day 3 (Table 1). Patients in the control arm received 2048 IU of HP-hMG vs 1900 IU in the experimental arm (*p* = 0.04). The number of transferable embryos was higher in the experimental arm (4.4 vs 3.6 in the control group, *p* = 0.0008). While there is a difference in the number of transferable embryos, the amount of top quality (EQ. 1) and high quality (EQ. 2) embryos is similar in all three patient groups (Table 1 and Supplementary Table 1). Histogram plots of the main patient characteristics (Figs. 2 and 3) show the three-dimensional and two-dimensional distributions and confirm the similarity between the two arms.

**Table 1:**
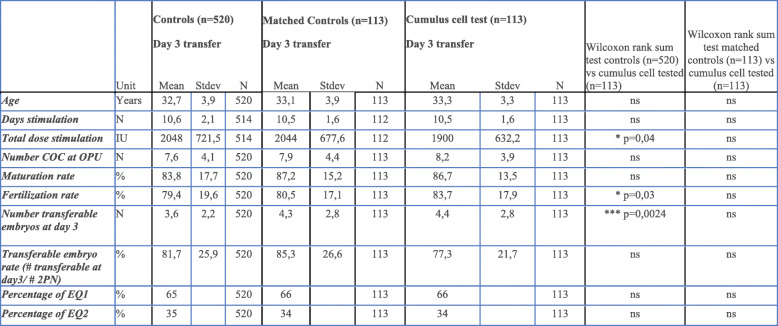
Characteristics of the Fresh ICSI Cycle in 113 cumulus cell tested patients, 520 control patients and 113 matched controls (for Kaplan-Meier analysis)

### Clinical pregnancy and live birth rates after fresh eSET

**Fig. 4:**
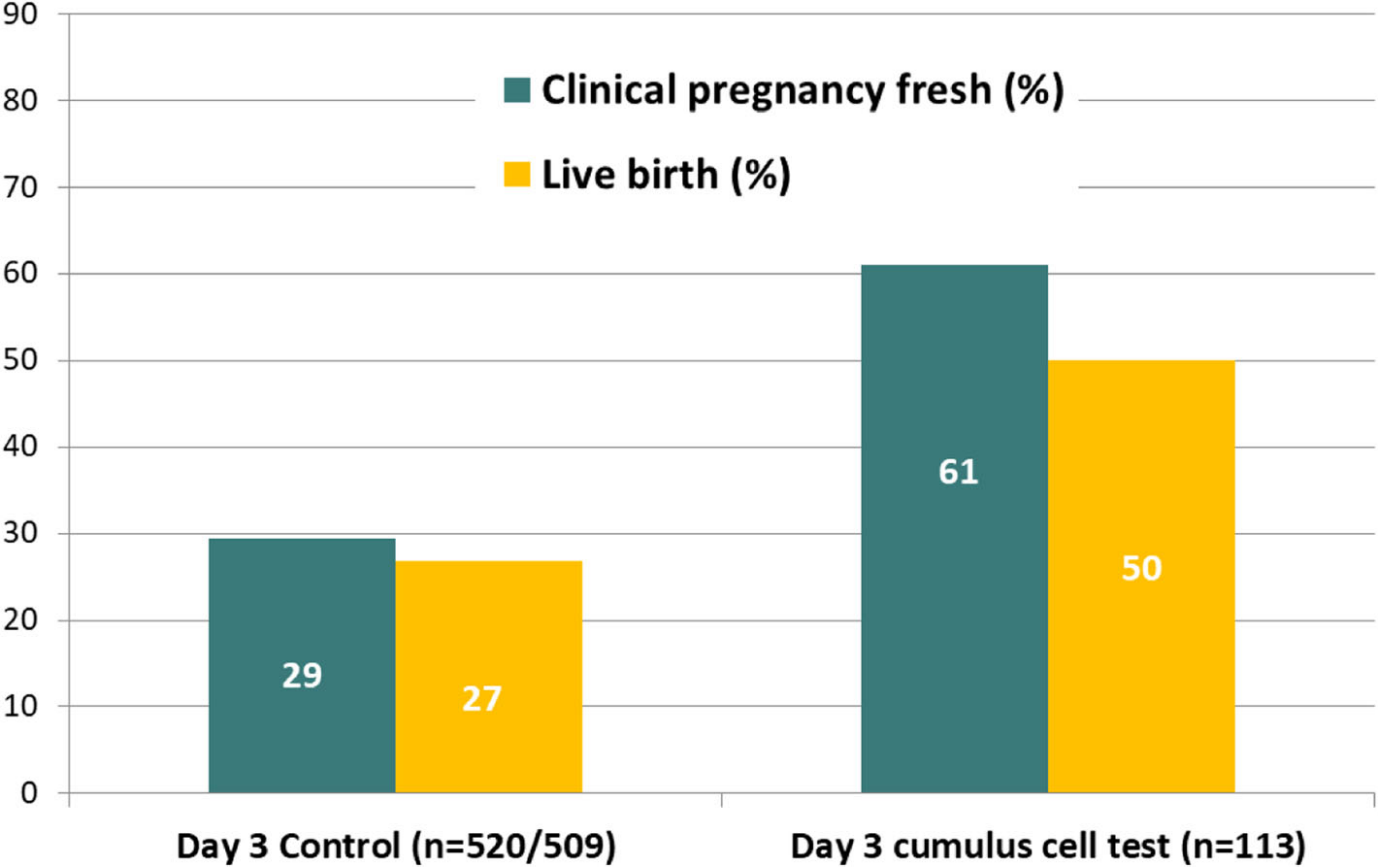
CPR and LBR after fresh Day 3 eSET in a prospective study with 113 patients in the experimental arm (with cumulus cell test) and 520 patients (520 patients for CPR and 509 patients for LBR) in the control arm (without the cumulus cell test) at Universitair Ziekenhuis Brussel. In the experimental arm Day 3 transfer is based on embryo morphology and the cumulus cell test (right side), and in the control arm on morphology only (left side). CPR is shown in blue bars and LBR in orange. Numbers in the bars are the % clinical pregnancies or live birth, respectively

The CPR was 69/113 (61.1%) in the experimental arm and 153/520 (29.4%, weighted average) in the control arm (*p* < 0.0001) (Fig. 4). Patients are subcategorized for age and number of transferable embryos (3 age and 3 embryo quality categories: a total of 9 categories). With the weighted average the proportion of each group is taken into account to calculate the weighted averages for the clinical outcomes. As such, the contribution of each of the 9 subcategories is equal in the test group and the control group.

The LBR in the experimental arm was 56/113 (49.6%) vs 135/509 (26.7%, weighted average) in the control arm (*p* < 0.0001) (Fig. 4). As far as LBR is concerned, no patients were lost to follow-up in the experimental arm, whereas eleven patients were lost to live birth follow-up in the control arm. The majority of the 56 deliveries in the experimental arm were singleton live-born neonates. Two pregnancies resulted in a monozygotic twin birth. There were no stillbirths.

### Subgroup analysis with respect to number of transferable embryos and age

The experimental arm was further analyzed with respect to the number of transferable embryos in three subgroups (2, 3–4 and > 4 transferable embryos) and with respect to age in two subgroups (younger than 35 years, and 35–39 years old).

**Fig. 5:**
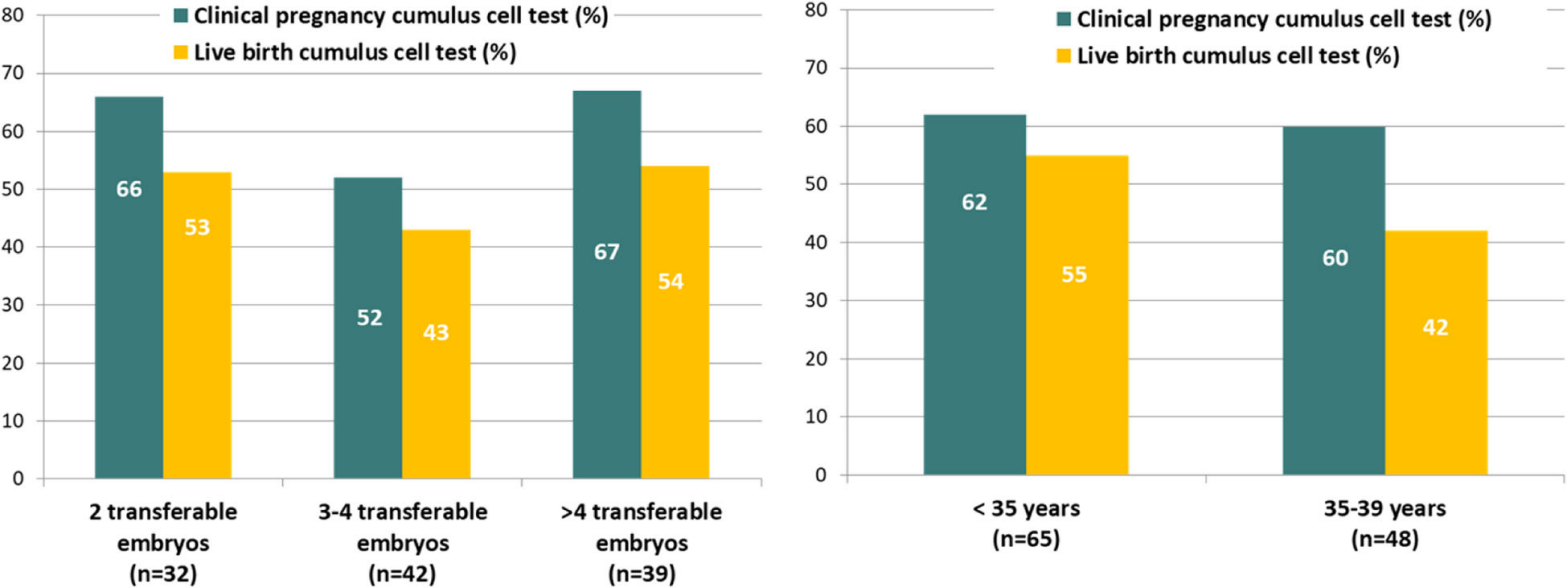
CPR and LBR after fresh Day 3 transfer in a prospective study with 113 patients in the experimental arm. CPR and LBR in the experimental arm (with cumulus cell test) are shown only in relation to the number of embryos of transferable quality (left side) and age groups (right side). CPR is shown in blue bars and LBR in orange. Numbers in the bars are the % clinical pregnancies or live birth respectively. Comparisons were performed using the Chi square analysis between the different subgroups and revealed no statistical difference

In the two subgroups with 2 and > 4 embryos, CPR was 66 and 67% and LBR was 53 and 54%, respectively. In the subgroup with 3–4 transferable embryos CPR and LBR dropped to 52% (*p* = 0,3) and 43% (*p* = 0,5), respectively. However, the CPR and LBR were not significantly lower (left side Fig. 5).

CPR was almost identical in both age subgroups (60% versus 62%; *p* = 0,9), while LBR was higher in the younger patient cohort versus the older patient cohort (55% versus 42%: *p* = 0,1), although this difference was not statistically significant.

### Time-to-pregnancy analysis

**Fig. 6:**
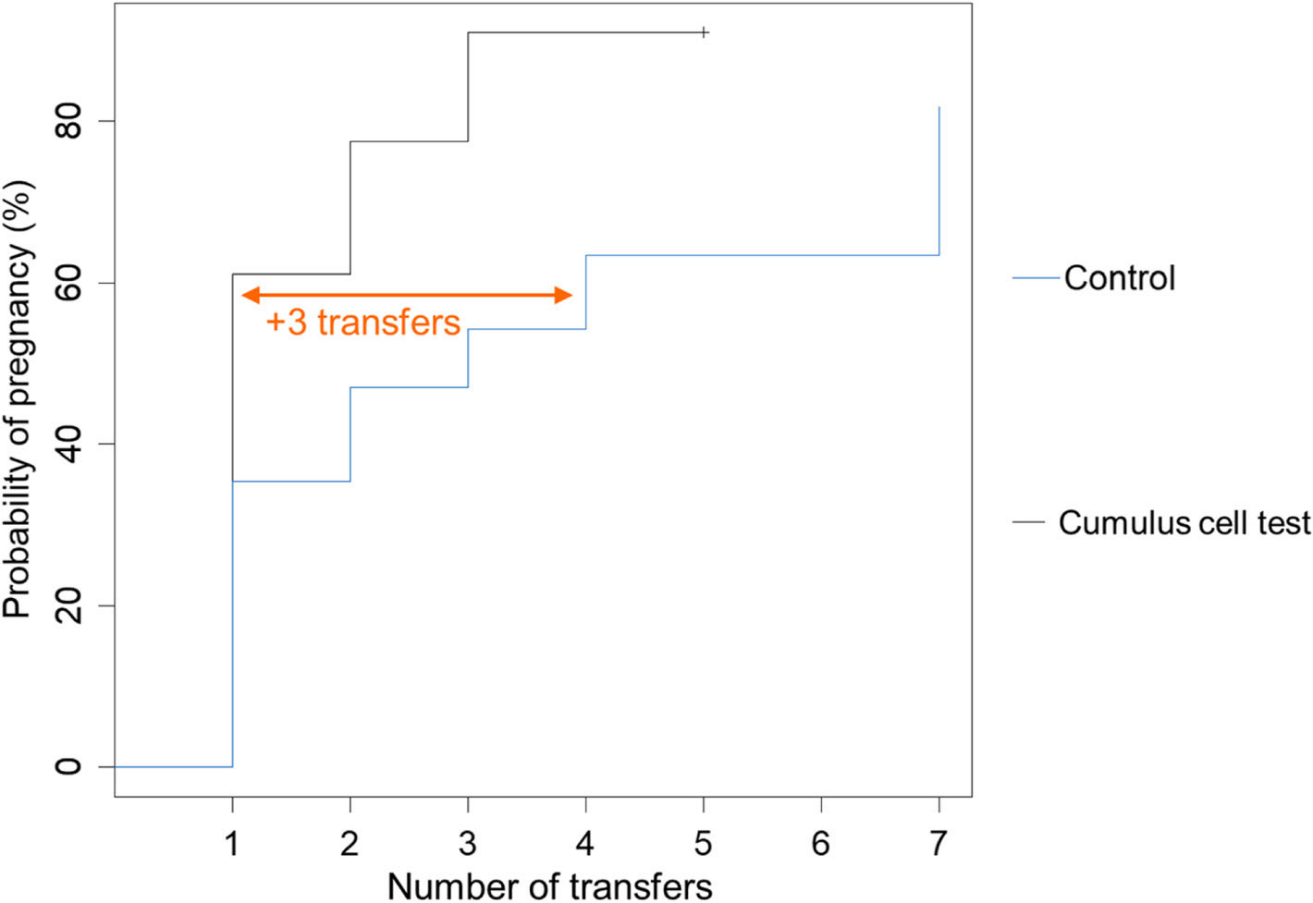
Kaplan-Meier curves for patients with > 2 transferable embryos on Day 3. Graph was generated on the data of 113 patients for each arm with 113 fresh transfers for each arm, plus 137 and 88 frozen embryo transfers for the control and the cumulus cell test arm respectively (451 single embryo transfers in total). 1 = fresh embryo transfer, 2–7 = frozen embryo transfers

Time-to-pregnancy was evaluated by Kaplan-Meier analysis for the 113 patients in the experimental arm vs 113 exact matched controls for all patients with > 2, or > 3, or > 4, or > 5, or > 6 transferable embryos. Time-to-pregnancy was significantly shorter in the experimental arm for all patients (*p* < 0.0001, Fig. 6). As an example, when considering all patients with at least two transferable embryos, three additional Day 3 transfers were needed in the control arm to achieve a clinical pregnancy compared with the patients in the experimental arm (Fig. 6).

## Discussion

This interventional non-randomized assessor-blinded cohort study evaluated the effect of a non-invasive cumulus cell test measuring oocyte quality in combination with morphology assessment on clinical outcome after fresh Day 3 eSET, as a method to improve the selection of the best embryo. In comparison to our earlier pilot study [1], the number of patients in the experimental arm was higher (*n* = 113 patients) and in the control arm (*n* = 520 patients) with routine morphological scoring only the number of patients was 4,7 times higher compared to the experimental arm. As a consequence, patients were analysed with a different statistical approach (a weighted generalized linear model) in comparison with the previous exact matched case-control study. Inclusion of a higher number of control patients allowed to obtain a more reliable evaluation of background CPR and LBR for Day 3 eSET over an increased time frame. Indeed, patient-to-patient variability in the heterogeneous infertile population over time could skew the results. A second asset of the current study was that all patients were treated with the same stimulation protocol (GnRH antagonist and HP-hMG), which was not the case in the pilot study.

Comparison of potential differences between the experimental and control arm showed two small but significant differences between patient characteristics. Patients in the experimental arm had received only 1.900 IU of HP-hMG and had on average 4.4 transferable embryos available at Day 3, while patients in the control arm received 2.048 IU of HP-hMG but had 3.6 embryos available for transfer at Day 3. About 40% of patients had only 2 transferable embryos on Day 3 in the control arm, in the experimental arm this group was 30%. Because of the potential bias induced by these differences, it was decided to evaluate CPR and LBR by stratifying for age and number of transferable embryos. Furthermore, fertilization rates are different (*p* = 0.03), but high in both arms. They comply with the key performance indicators of normal fertilization with ICSI (competence ≥65%; benchmark ≥80%, Vienna consensus, [17]).

Subgroup analysis showed that age had no influence on the clinical pregnancy rate when choosing for the first embryo to transfer in the fresh cycle with the cumulus cell test. Also, the LBR was not significantly different in both age groups. However, sample numbers in both age groups are rather low, and there seems to be a trend towards lower LBR in the older age group.

It seems intuitive that the benefit of the cumulus cell test would be largest for patients with many oocytes, and that the value would be rather limited when only two transferable embryos of similar morphological quality are available. Subgroup analysis in patients with 2, 3–4 and > 4 embryos available on Day 3 showed no statistically significant difference in outcome between the subgroups, suggesting that the test enables the selection of the best oocyte independent of ovarian response.

The study has several limitations. The cumulus cell test genes are currently only validated for patients who receive ovarian stimulation with HP-hMG in a GnRH antagonist protocol. It was already reported earlier that the expression of selected genes in cumulus cells differ depending on the type of gonadotrophins used [2, 21].

Furthermore, this single center study is an interventional non-randomized assessor-blinded cohort study and not a randomized trial. Results from a multicenter RCT would yield a higher level of evidence.

In the embryology laboratory, the embryologists and lab technicians had to perform individual denudation of all the oocytes. On average this took 15–30 min extra working time for a maximum of 16 oocytes per cumulus cell tested case. On the other hand, the cumulus cell test received a high acceptance rate by the patients eligible for the study, when explained by the clinicians.

This study ran over a long period of time. This is principally due to the large number of clinical studies performed in parallel at the ART center of Universitair Ziekenhuis Brussel and the fact that the majority of patients seeking fertility treatment were stimulated with other stimulation regimes than HP-hMG, had a blastocyst transfer on Day 5 or underwent a freeze-all procedure.

This study was done for Day 3 transfers only. Today many centers prefer a Day 5 transfer. However, for patients with a low oocyte yield, a Day 5 transfer policy increases the risk of having no embryo available for transfer [39]. In ART centers that routinely apply Day 3 fresh and Day 5 fresh and frozen transfers, women with higher number of embryos (generally 4 or more) may be advised to have a Day 5 transfer, while women with fewer embryos would be directed to a Day 3 transfer.

Our data with patients having two to 17 transferable embryos suggest that a Day 3 eSET, when implementing the cumulus cell test, may result in similar pregnancy rates compared to a Day 5 eSET (by conventional morphological selection). Whether combining cumulus cell testing with a Day 5 transfer regime could further increase the efficiency is as yet unknown and under current investigation.

## Conclusions

The study reached its primary endpoint: in the experimental arm the CPR was 30% higher than in the control arm (from 29 to 61%). The LBR (secondary endpoint) increased by 23% from 27 to 50% in the experimental arm vs the control arm. From the patients in the experimental arm who were either pregnant or had all their embryos transferred cumulatively, the Kaplan-Meier calculations showed a significant reduction of 3 transfer cycles versus the control arm to achieve a clinical pregnancy.

In summary, this study provided further evidence of the clinical validity of the non-invasive cumulus cell test in Day 3 eSET. Multicenter randomized studies are underway to evaluate the validity in Day 5 eSET in fresh and frozen embryo transfers and to determine cost-efficiency of non-invasive embryo selection.

## Supplementary Information

**Additional file 1: Supplementary Table 1.** Fraction of patients with EQ 1 and EQ 2 embryos in the control arm (*n* = 520), the experimental arm with the cumulus cell test (*n* = 113) and the exact matched controls (for Kaplan-Meier analysis) (*n* = 113). Comparisons were performed using the Chi square analysis between the different subgroups and revealed no statistical difference.

**Additional file 2: Supplementary Table 2.** Infertility indication in both arms (male, female or mixed cause of infertility). Comparisons were performed using the Chi square analysis between the different arms and revealed no statistical difference.

## Data Availability

The data that support the findings of this study are available from Fertiga NV but restrictions apply to the availability of these data, which were used under license for the current study, and so are not publicly available. Anonymized clinical data and ranking data are available on reasonable request following the GDPR guidelines, with permission of Fertiga NV and Centre for Reproductive Medicine, Universitair Ziekenhuis Brussel.

## Abbreviations

CPR: Clinical pregnancy rate
eSET: Elective single embryo transfer
LBR: Live birth rate
ICSI: Intra-cytoplasmic sperm injection
PCOS: Polycystic ovary syndrome
HP-hMG: Highly purified human menopausal Gonadotropin
GnRH: Gonadotropin releasing hormone
qRT-PCR: Quantitative reverse transcriptase polymerase chain reaction
ART: Assisted reproductive technology
IVF: In vitro fertilization
CC: Cumulus cells
SET: Single embryo transfer
DET: Double embryo transfer
OPR: Ongoing clinical pregnancy rate
PGT-A: Pre-implantation genetic testing for aneuploidy
EQ: Embryo quality
B2M: Beta-2-Microglobulin
UBC: Ubiquitin C
EFNB2: Ephrin B2
CAMK1D: Calcium/Calmodulin Dependent Protein Kinase ID
SASH1: SAM And SH3 Domain Containing 1
