# Supplementary material for: Improved clinical outcomes after non-invasive oocyte selection and Day 3 eSET in ICSI patients
Source: Reprod Biol Endocrinol. 2021 Feb 19;19:26. doi: 10.1186/s12958-021-00704-5 (PMC7892761; doi:10.1186/s12958-021-00704-5)
Supplement: Supplementary file 1 — Additional file 1: Supplementary Table 1. Fraction of patients with EQ 1 and EQ 2 embryos in the control arm (n = 520), the experimental arm with the cumulus cell test (n = 113) and the exact matched controls (for Kaplan-Meier analysis) (n = 113). Comparisons were performed using the Chi square analysis between the different subgroups and revealed no statistical difference. [file 12958_2021_704_MOESM1_ESM.docx]

Supplementary Table 1. Fraction of patients with EQ 1 and EQ 2 embryos in the control arm (n=520), the experimental arm with the cumulus cell test (n=113) and the exact matched controls (for Kaplan-Meier analysis) (n=113). Comparisons were performed using the Chi square analysis between the different subgroups and revealed no statistical difference.

|  |  | *Fraction of patients with* | | |
| --- | --- | --- | --- | --- |
|  | *Total # patients* | *Only EQ 1 embryos* | *Only EQ 2 embryos* | *EQ 1 + EQ 2 embryos* |
| **Controls** | 520 | 179 (34%) | 65 (13%) | 276 (53%) |
| **Cumulus cell test** | 113 | 32 (28%) | 15 (13%) | 66 (58%) |
| **Exact matched (Kaplan-Meier) controls** | 113 | 36 (32%) | 16 (14%) | 61 (54%) |
