# Supplementary material for: Improved clinical outcomes after non-invasive oocyte selection and Day 3 eSET in ICSI patients
Source: Reprod Biol Endocrinol. 2021 Feb 19;19:26. doi: 10.1186/s12958-021-00704-5 (PMC7892761; doi:10.1186/s12958-021-00704-5)
Supplement: Supplementary file 2 — Additional file 2: Supplementary Table 2. Infertility indication in both arms (male, female or mixed cause of infertility). Comparisons were performed using the Chi square analysis between the different arms and revealed no statistical difference. [file 12958_2021_704_MOESM2_ESM.docx]

Supplementary Table 2. Infertility indication in both arms (male, female or mixed cause of infertility). Comparisons were performed using the Chi square analysis between the different arms and revealed no statistical difference.

| **Cause of infertility** | **Cumulus cell tested n=113** | **Control n=113** | **Control n=520** | **Chi-square Test** |
| --- | --- | --- | --- | --- |
| **Male** | 34% | 41% | 38% | ns |
| **Female** | 55% | 53% | 50% | ns |
| **Mixed** | 11% | 6% | 12% | ns |
